# The *Drosophila melanogaster* CHD1 Chromatin Remodeling Factor Modulates Global Chromosome Structure and Counteracts HP1a and H3K9me2

**DOI:** 10.1371/journal.pone.0059496

**Published:** 2013-03-22

**Authors:** Lakshmi Bugga, Ivy E. McDaniel, Liana Engie, Jennifer A. Armstrong

**Affiliations:** W.M. Keck Science Department, Claremont McKenna College, Pitzer College, Scripps College, Claremont, California, United States of America; University College London, United Kingdom

## Abstract

CHD1 is a conserved chromatin remodeling factor that localizes to active genes and functions in nucleosome assembly and positioning as well as histone turnover. Mouse CHD1 is required for the maintenance of stem cell pluripotency while human CHD1 may function as a tumor suppressor. To investigate the action of CHD1 on higher order chromatin structure in differentiated cells, we examined the consequences of loss of CHD1 and over-expression of CHD1 on polytene chromosomes from salivary glands of third instar *Drosophila melanogaster* larvae. We observed that chromosome structure is sensitive to the amount of this remodeler. Loss of CHD1 resulted in alterations of chromosome structure and an increase in the heterochromatin protein HP1a, while over-expression of CHD1 disrupted higher order chromatin structure and caused a decrease in levels of HP1a. Over-expression of an ATPase inactive form of CHD1 did not result in severe chromosomal defects, suggesting that the ATPase activity is required for this *in vivo* phenotype. Interestingly, changes in CHD1 protein levels did not correlate with changes in the levels of the euchromatin mark H3K4me3 or elongating RNA Polymerase II. Thus, while CHD1 is localized to transcriptionally active regions of the genome, it can function to alter the levels of HP1a, perhaps through changes in methylation of H3K9.

## Introduction

In eukaryotes, DNA is assembled with histones and other proteins to form chromatin. Genome-wide localization studies of chromatin proteins and histone modifications in *Drosophila* tissue culture cells have identified a number of chromatin states (from 5 to 30) that describe the status of chromatin relative to gene activity, cis-acting sequences, and dosage compensation [1], [2]. To complicate matters, the status of chromatin is not static, undergoing dynamic changes during gene expression, DNA repair, DNA replication and other processes. Proteins responsible for orchestrating these changes include histone modifying enzymes that acetylate, methylate, phosphorylate and ubiquitylate specific residues; and ATP-dependent chromatin remodeling factors, which are involved in assembling, repositioning, evicting, and unwrapping nucleosomes [3], [4]. ATP-dependent chromatin remodelers are members of the SNF2 family, which can be divided into 24 subfamilies (often named after their founding members), a subset of which has been implicated in chromatin remodeling [5], [6]. In *Drosophila*, a number of chromatin remodeling factors are localized to active genes, including Brahma (BRM), Kismet (KIS), CHD1 (chromodomains, helicase/ATPase domain, DNA binding domain) and dMi-2 [7]–[10]. Both *brm* (a SWI/SNF subfamily member) and *kis* (a CHD subfamily member) were identified as trithorax group genes [11]–[13], while *chd1* and *dMi-2* (both CHD subfamily members) have not displayed mutant phenotypes that would characterize them as trithorax group genes [14], [15]. The chromatin remodelers facilitate different stages of transcription. BRM is necessary for the binding of the initiating form of Pol II [7], while KIS is needed for the transition from the promoter clearance form of Pol II (Pol IIo^ser5^) to the elongating form Pol IIo^ser2^ [8].

CHD1 is a critical protein in several transcriptional processes from initiation to termination [16]. *In vitro* studies have found that human CHD1 associates with the pre-initiation transcription complex through interactions with Mediator [17]. Yeast Chd1 was identified as a factor required for remodeling the nucleosomal *PHO5* promoter and for transcriptional activation of the gene [18]. CHD1 co-localizes with elongation factors and elongating RNA Polymerase II in flies and yeast [8], [19], [20]. Yeast Chd1 is essential for chromatin structure at the 3′ ends of genes, and transcriptional termination fails to occur in its absence [21]. Consistent with a role for Chd1 in maintaining chromatin structure at active genes, transcription initiation from cryptic promoters occurs in yeast in the absence of Chd1 [22], [23], and Chd1 is critical for genome-wide nucleosomal positioning over gene bodies [24].

CHD1 is a chromatin remodeler of particular interest in that it has been implicated in multiple biological processes. In mice it is essential for embryonic stem (ES) cell pluripotency and the formation of induced pluripotent stem (iPS) cells [25]. Without the CHD1 remodeler, an increase in the heterochromatic mark H3K9me3 was observed, and the ES cells displayed a tendency towards neuronal differentiation. Recent studies have implicated CHD1 as a tumor suppressor protein. Deletion or mutation of one copy of CHD1 was found associated with prostate cancer, with cells lacking CHD1 displaying an increase in invasiveness [26], [27]. Loss of CHD1 did not affect expression of genes known to encode factors important for invasion, and thus it is unclear how CHD1 functions to inhibit invasion. Given these roles in stem cell and cancer biology, understanding the mechanism of action of CHD1 is of high importance.

*Drosophila* provides an ideal model organism in which to study the function of CHD1. Unlike many genes encoding chromatin remodeling factors in *Drosophila*, *chd1* is not essential for life [15], [28]. Instead, *chd1* is required for wing development and male and female fertility [15]. Although CHD1 co-localizes with elongating RNA Polymerase II (Pol II) at active genes on *Drosophila* polytene chromosomes [8], CHD1 is not required for the presence of elongating Pol II on polytene chromosomes [15]. Loss of CHD1 results in a decrease in the incorporation of the H3.3 histone variant in sperm chromatin following fertilization [28] and a reduction in replication-independent H3.3 deposition in polytene chromosomes [29], suggesting that CHD1 is important for nucleosome stability at active genes. While much can be learned from examining chromatin at the nucleosomal level, little is known about the effects of chromatin remodeling factors on higher order chromatin structure. To address this question, we examined the function of CHD1 on polytene chromosomes from salivary glands of third instar larvae. Polytene chromosomes of *Drosophila* allow us to directly visualize changes in higher order chromatin structure in transcriptionally active interphase cells in a way not possible in diploid tissues or tissue culture cells. In this study, we found that the CHD1 remodeler is critical for normal chromosome morphology, as either loss of *chd1* or over-expression of *chd1* resulted in defects in chromosome structure, with CHD1 levels inversely proportional to levels of HP1a and H3K9me2. These findings implicate the CHD1 ATPase in the maintenance of global chromosome structure and the regulation of heterochromatic elements in differentiated cells.

## Results

### The Chromatin Remodeler CHD1 is Required for Global Chromosome Structure

**Figure 1:**
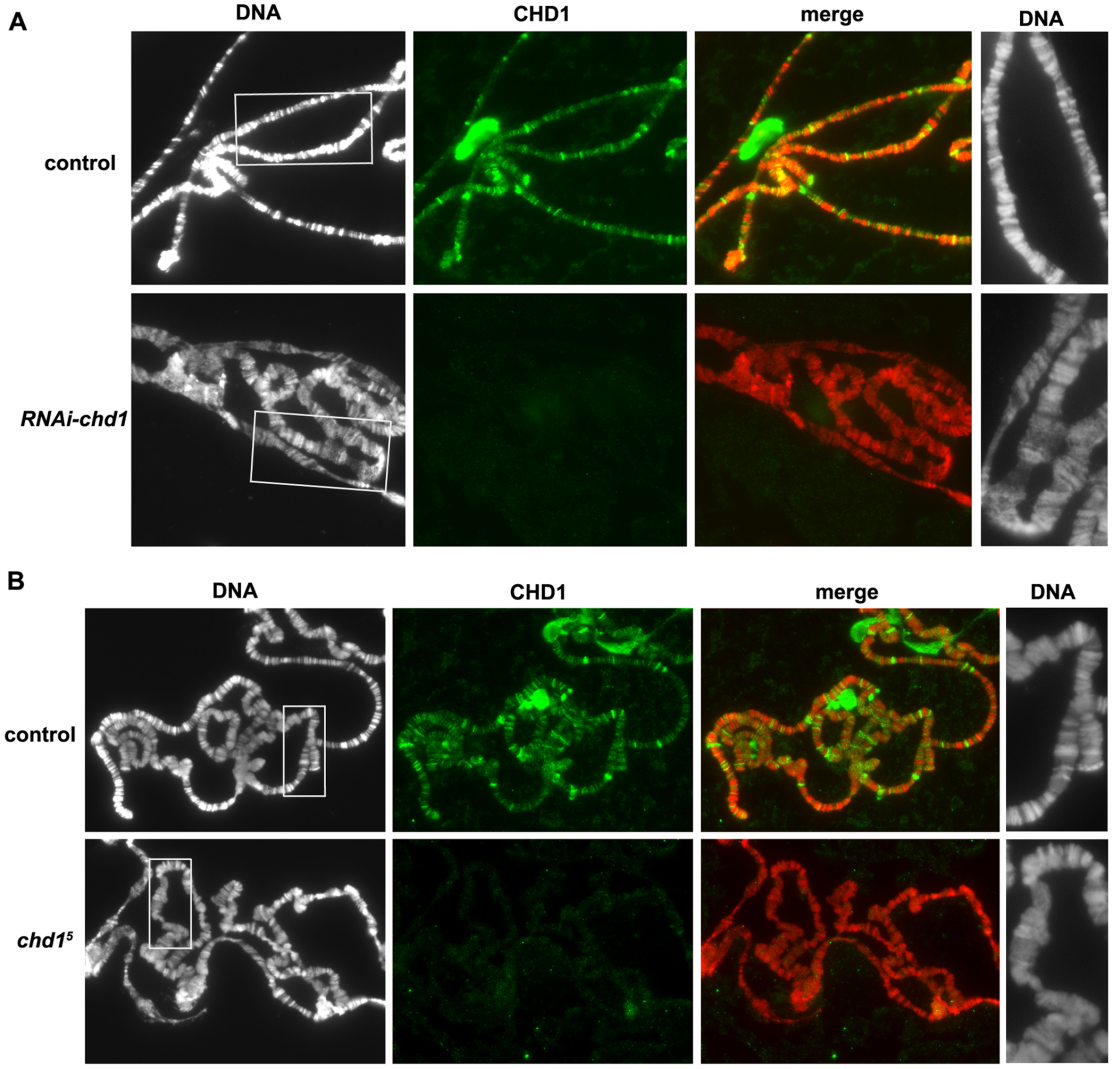
Loss of CHD1 results in defects in the structure of polytene chromosomes. (A) Polytene chromosomes derived from salivary glands expressing *chd1* hp RNA display morphological defects. Control is *AB1-gal4/UAS-gfp,* and *RNAi-chd1* is *VDRC103640/+; AB1-gal4/+.* (B) *chd1* mutant larvae raised at 18°C display more subtle disruptions in polytene structure. Control chromosomes were prepared from flies that underwent a precise excision of the same P element that was imprecisely excised to generate the *chd1^5^* null allele [15]. Chromosomes were stained with DAPI (white in left panel, red in merge) and immunostained with anti-CHD1 (green) as described [7]. Magnified views of a portion of the chromosomes are shown in the right column.

**Figure 2:**
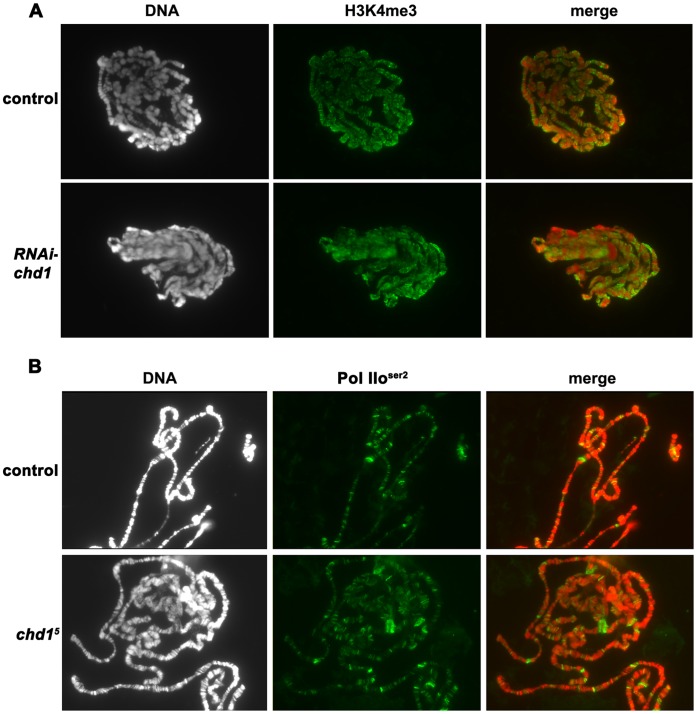
Loss of CHD1 does not affect H3K4me3 or elongating RNA Polymerase II. (A) Polytenes derived from *UAS-gfp/actin5C-gal4* (control) larvae and *VDRC26277/+; +/actin5C-gal4* larvae (*RNAi-chd1*) show similar levels of the transcriptionally active mark H3K4me3. Chromosomes were stained with DAPI (white in left panel, red in merge) and immunostained with anti-H3K4me3 (green) as described [40]. (B) Elongating RNA Polymerase II persists on chromosomes lacking CHD1. Control chromosomes were prepared from flies that underwent a precise excision of the same P element that was imprecisely excised to generate the *chd1^5^* null allele [15]. Chromosomes were stained with DAPI (white in left panel, red in merge) and immunostained with anti-Pol IIo^ser2^ (green) as described [7].

**Figure 3:**
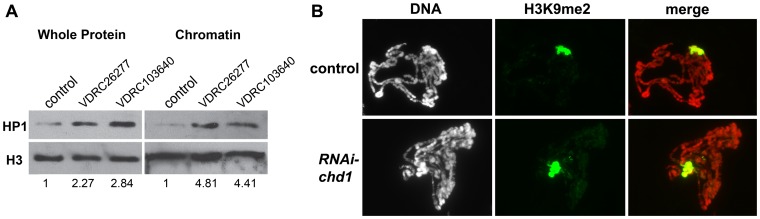
Loss of CHD1 results in an increase in levels of HP1a and H3K9me2. (A) Western blot analysis reveals that loss of CHD1 by hpRNA results in a 4.8-fold increase in the ratio of chromatin-bound HP1a to chromatin-bound histone H3. Control is *UAS-lacZ/AB1-gal4*. *chd1* loss of function polytenes were derived from *VDCR26277/+; AB1-gal4/+ and VDRC103640/+; AB1-gal4/+* larvae. (B) Loss of CHD1 results in an increase in levels of H3K9me2. Control is *UAS-lacZ/AB1-gal4 and RNAi-chd1* is *VDRC26277/+; AB1-gal4/+.* Chromosomes were stained with DAPI (white in left panel, red in merge) and immunostained with anti-H3K9me2 (green) as described [40].

In mouse ES cells, CHD1 maintains open chromatin by preventing the spread of repressive chromatin marks, thereby maintaining pluripotency and preventing differentiation [25]. It is unknown if this activity is conserved across species, and/or if is restricted to ES cells. If *Drosophila* CHD1 maintains an open chromatin state in differentiated cells, we would predict that loss of *chd1* might lead to an increase in chromosome condensation. Polytene chromosomes derived from *chd1* null mutant larvae appeared normal in overall morphology [15], however maternally contributed CHD1 may have masked chromosomal mutant phenotypes. In an attempt to overcome the persistence of maternally contributed *chd1* message and to test the hypothesis that CHD1 counteracts the spreading of transcriptionally repressive chromatin, we made use of two distinct GAL4-inducible RNA interference (RNAi) transgenic lines generated by the Vienna *Drosophila* RNAi Center (VDRC) [30]. RNAi *Drosophila* lines can be problematic in that they may display off-target activity. BLAST searches suggested that no off-targets are likely for either of the two RNAi sequences. Moreover, as the two hairpin RNAs (hpRNAs) target distinct, non-overlapping regions of the *chd1* transcript (Figure S1B), we propose that any shared phenotypes are due to loss of *chd1*. Expression of the *chd1* hpRNA in salivary glands resulted in a substantial reduction in CHD1 levels on polytene chromosomes (Figure 1A). Rather than an increase in chromosome condensation as predicted, we observed chromosomal phenotypes that ranged from normal morphology [29] to disruption of normal banding patterns with overall alterations of chromosome structure (see Figures 1A, 2A, 3B, S1A, Figures S4). An analysis of three independent experiments revealed that 57% of polytene spreads from larvae expressing *chd1* hpRNA (that showed reduced levels of CHD1 by immunofluorescence) displayed a disrupted chromosomal phenotype (N = 53), while none of the polytene chromosomes from age-matched control larvae displayed this phenotype (N = 27). We observed the disrupted chromosomal phenotype using formaldehyde/acetic acid-based fixatives as well as a citric acid-based fixative. We failed to observe defects in chromosome structure following expression of hpRNA directed against *Spt5* (Figure S2), suggesting that this phenotype is not a consequence of activating the RNAi pathway in salivary glands.

Since loss of CHD1 by RNAi resulted in disrupted polytene chromosomes, we reasoned that we should observe a similar phenotype in our *chd1* null mutant animals, provided that we could overcome the problem of maternal purdurance. By raising *chd1* mutant larvae at 18°C and by looking at oldest possible third instar larvae (18 to 20 days old), we observed a less severe, but similar chromosomal phenotype and a loss of precise banding (Figure 1B, 2B). Analysis of seven independent experiments revealed that 47% of polytene chromosome spreads from *chd1* mutant larvae (N = 68) displayed chromosomal defects as compared to 11% of polytene spreads from age-matched control larvae (N = 66).

We note that both the severity of defects as well as their presence or absence is variable in larvae with reduced levels of CHD1 protein. In each experiment, the control and experimental larvae were staged for each of the experiments such that crosses were begun on the same day at the same temperature, and parents were “flipped” to fresh bottles for a 24-hour time period to obtain developmentally staged larvae. To account for any differences in the development of different genotypes, third instar larvae were staged by the status of their anterior spiracles. We propose that the variability in chromosomal defects is due to variability in maternal purdurance of CHD1 protein, different fixation conditions in different experiments, and the precise developmental stage of larvae.

Chromatin remodeling factors Ino80 and Isw2 have been found to function at replication forks in yeast [31] to attenuate activation of the S phase checkpoint in response to replication stress [32]. It is possible that our observed defects in polytene chromosomes lacking CHD1 are a consequence of defects in DNA replication. To address this question, we analyzed DNA levels of polytene squashes that represented intact chromosome spreads (in which all chromosomes were present in the image). We observed no significant differences in DAPI staining in chromosomes derived from salivary glands lacking CHD1 as compared to control chromosomes (Figure S3A). Thus, a disruption of polytene structure and normal banding patterns resulting from loss of CHD1 are not a consequence of changes in DNA levels, indicating that one of the functions of CHD1 in somatic cells is to maintain global chromosome structure.

Levels of the histone variant H3.3 are reduced on live and fixed polytene chromosomes derived from *chd1* mutant larvae or larvae expressing *chd1* hpRNA, indicating that the remodeler is required for deposition and/or stabilization of this histone variant [29]. H3.3 is enriched in active genes and enriched for euchromatin-typical histone modifications, including H3K4me3 [33]. To investigate the effects of loss of CHD1 on euchromatin, we examined H3K4me3 and observed no global changes in the levels of this mark on polytene chromosomes derived from larvae with reduced CHD1 (Figure 2A), consistent with prior observations [34]. CHD1 co-localizes with the elongating form of RNA Polymerase II (RNA Pol IIo^ser2^) [8], suggesting that CHD1 may be important for chromatin disruption or reassembly during transcription. We had previously observed that elongating RNA Polymerase II could persist in *chd1* mutant animals [15], however it is possible that maternal purdurance was masking a requirement for CHD1. We continue to observe Pol IIo^ser2^ on chromosomes derived from *chd1* mutant larvae grown at 18°C (Figure 2B). Thus, despite the localization of CHD1 to transcriptionally active genes and an involvement in H3.3 deposition and/or stabilization, this chromatin remodeler does not appear to be required for elongation or maintenance of histone modifications associated with euchromatin.

### Loss of CHD1 Increases Levels of HP1a and H3K9me2

The disrupted polytene chromosomes from larvae lacking CHD1 suggest that the remodeler alters higher order chromatin structure. To investigate whether CHD1 influences repressive chromatin, we examined the total levels of HP1a (heterochromatin protein 1a) in salivary glands expressing *chd1* hpRNA. HP1a binds H3 methylated on lysine 9 and is localized to telomeres, some euchromatic sites, and the chromocenter of polytene chromosomes (where the pericentric heterochromatin of the chromosomes coalesce) [35]. Loss of CHD1 resulted in a 2.3 to 2.8-fold increase in total HP1a protein (relative to levels of total H3), and a 4.4 to 4.8-fold increase in chromatin-bound HP1a (relative to levels of chromatin-bound H3) (Figure 3A). We similarly observed an increase in the levels of HP1a by immunofluorescence of polytene chromosomes (Figure S4). We asked whether loss of *chd1* altered the levels of H3K9me2 on polytenes, and found that levels of H3K9me2 increased on polytene chromosomes, with increases in H3K9me2 levels seen at the chromocenter and along the chromosome arms (Figure 3B). Thus, despite causing chromosomes to superficially appear more decondensed, loss of *chd1* results in an increase in HP1a and the H3K9me2 repressive mark.

### Over-expression of CHD1 Leads to Chromosomal Defects

**Figure 4:**
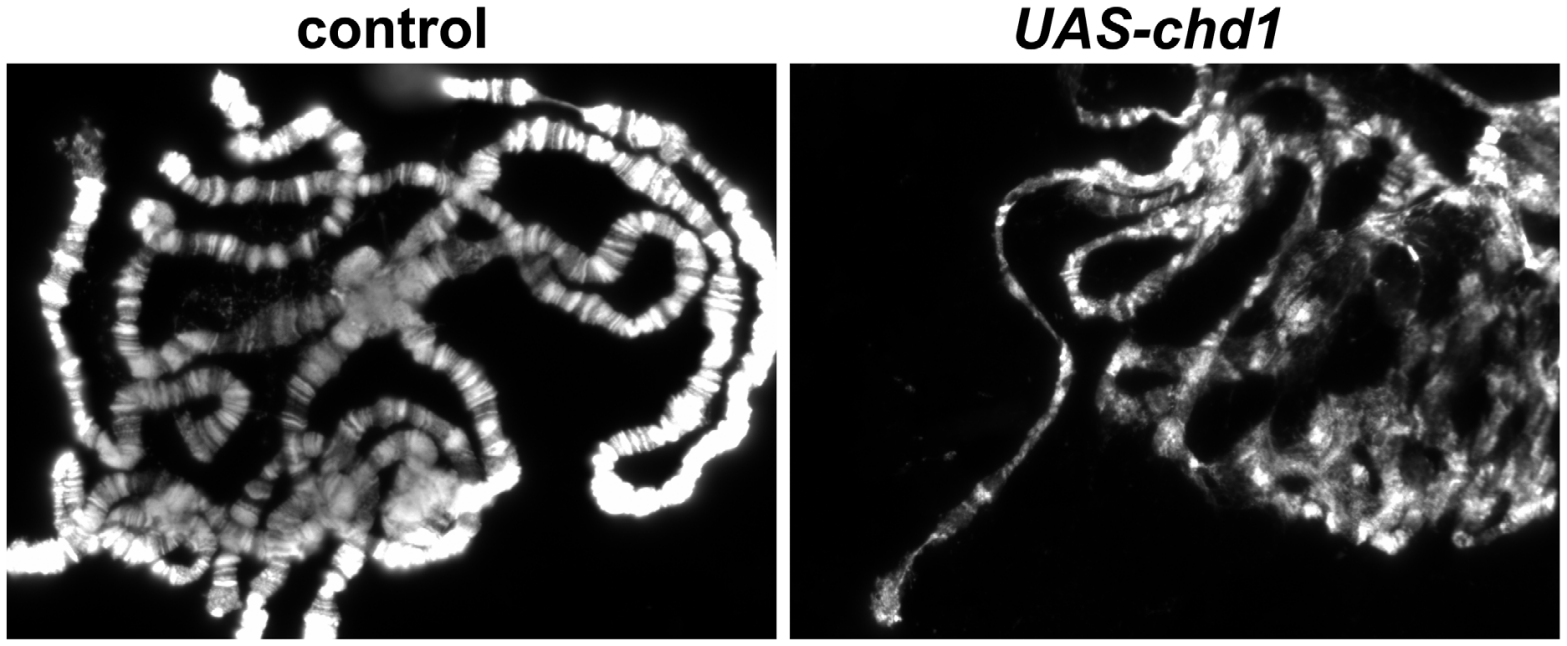
CHD1 over-expression results in polytene chromosomes with a disrupted structure. Polytenes prepared from *+/ey-GAL4; UAS-chd1^+^/+* larvae (*UAS-chd1*) are malformed, *+/ey-GAL4; UAS-gfp/+* (control) larvae show normal morphology. While *ey-GAL4* is an eye driver, it also expresses GAL4 in the salivary gland. DNA is stained with DAPI.

To further investigate the role of CHD1 in chromatin structure, we examined the consequences of over-expression of CHD1 on polytenes. We observed severe defects in polytene chromosomes following over-expression of *chd1* (from a cDNA construct) using a variety of different GAL4 drivers including *eyeless-GAL4*, *actin 5C-GAL4*, and *AB1-gal4* (Figure 4, S3C, S5). Some regions of the polytene chromosomes were thinner than expected, while other areas have lost integrity and appeared to be pulling apart. Over-expression of an ATPase inactive form of the remodeler (CHD1^KR^), in which a conserved lysine at position 559 is mutated to an arginine, did not result in severe chromosomal defects, despite similar protein levels on polytene chromosomes (Figures S3C, S5 and S6). An analysis of three independent experiments revealed that abnormal phenotypes were observed in 95% of polytene spreads from larvae over-expressing *chd1* (N = 57), 23% of polytene spreads from larvae over-expressing *chd1^KR^* (N = 55), and 2% of polytene chromosomes from age-matched control larvae over-expressing *lacZ* (N = 51). Thus, while the ATPase activity of CHD1 is important for modulation of global chromosome structure, over-expression of CHD1^KR^ can cause occasional defects in chromosome structure.

To determine whether over-expression of wild type CHD1 affects endoreplication, we analyzed DAPI levels of chromosomes that represented intact polytene squashes and found that DNA content was reduced by approximately 60% (Figures S3B and S3C). Thus, the chromosomal defects observed may be a consequence of disruptions in DNA replication. Alternatively, the reduction of DNA content could reflect slower development. Indeed, over-expression of *chd1* results in salivary glands that are smaller in size than control glands.

### HP1a and the H3K9me2 Heterochromatin Mark are Reduced on Chromosomes Over-expressing CHD1

**Figure 5:**
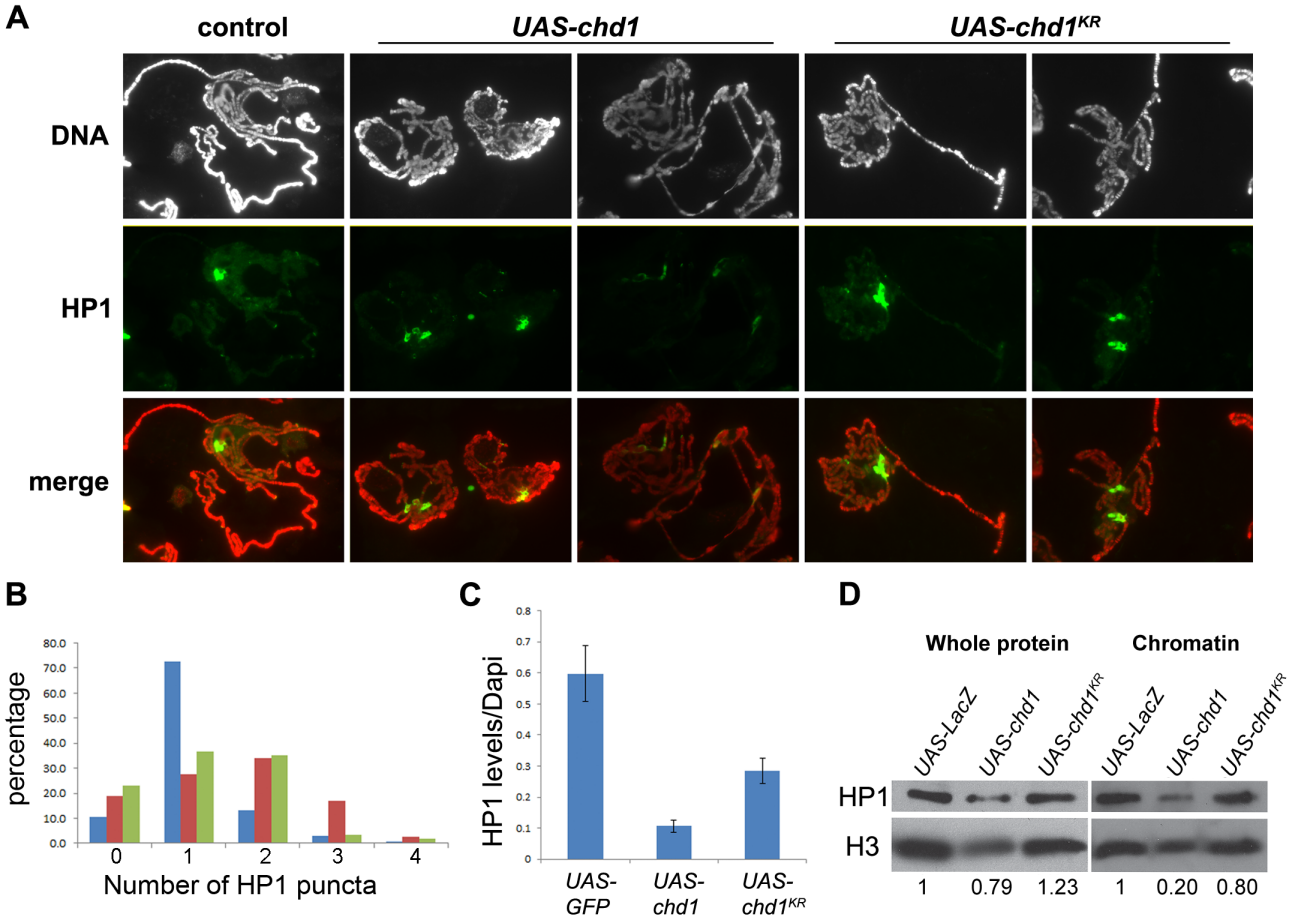
HP1a protein levels are reduced and its localization pattern is altered by over-expression of CHD1. (A) Polytene chromosomes derived from larvae over-expressing wild type CHD1 (*UAS-chd1*/*AB1-gal4*) show changes in distributions of HP1a puncta and reduced protein levels as compared to controls (*UAS-gfp/AB1-gal4*). Chromosomes derived from larvae overexpressing an ATPase inactive form of CHD1 (*UAS-chd1^KR^/AB1-gal4*) show an intermediate phenotype with levels of HP1a comparable to controls, and with two or more puncta of HP1a occasionally seen. Chromosomes were stained with DAPI (white in top panel, red in merge) and immunostained with anti-HP1a (green) as described [51]. (B) The number of HP1a puncta per single set of chromosomes (chromosomes were scored as one set if they clearly represented one nuclei) were counted blind (the individual scoring the results had not done the experiments and was blind to the genotypes of the chromosomes). Over-expression of CHD1 results in an increase in the number of HP1a puncta per set of chromosomes, *UAS-lacZ or UAS-gfp* controls (blue, N = 135), *UAS-chd1* (red, N = 153), *UAS-chd1^KR^* (green, N = 117). (C) The amount of HP1a on polytene chromosomes relative to DAPI intensity decreased following over-expression of CHD1. Data represent two independent experiments. HP1a levels are significantly lower following over-expression of CHD1 (P = 0.000014, Student’s t-test). Over-expression of CHD1^KR^ results in a more moderate reduction of HP1a levels (P = 0.0037). (D) Western blot analysis reveals that over-expression of CHD1 results in a 5-fold decrease in chromatin-bound HP1a (as measured as a ratio of HP1a to H3 to account for changes in endoreplication or salivary gland size).

**Figure 6:**
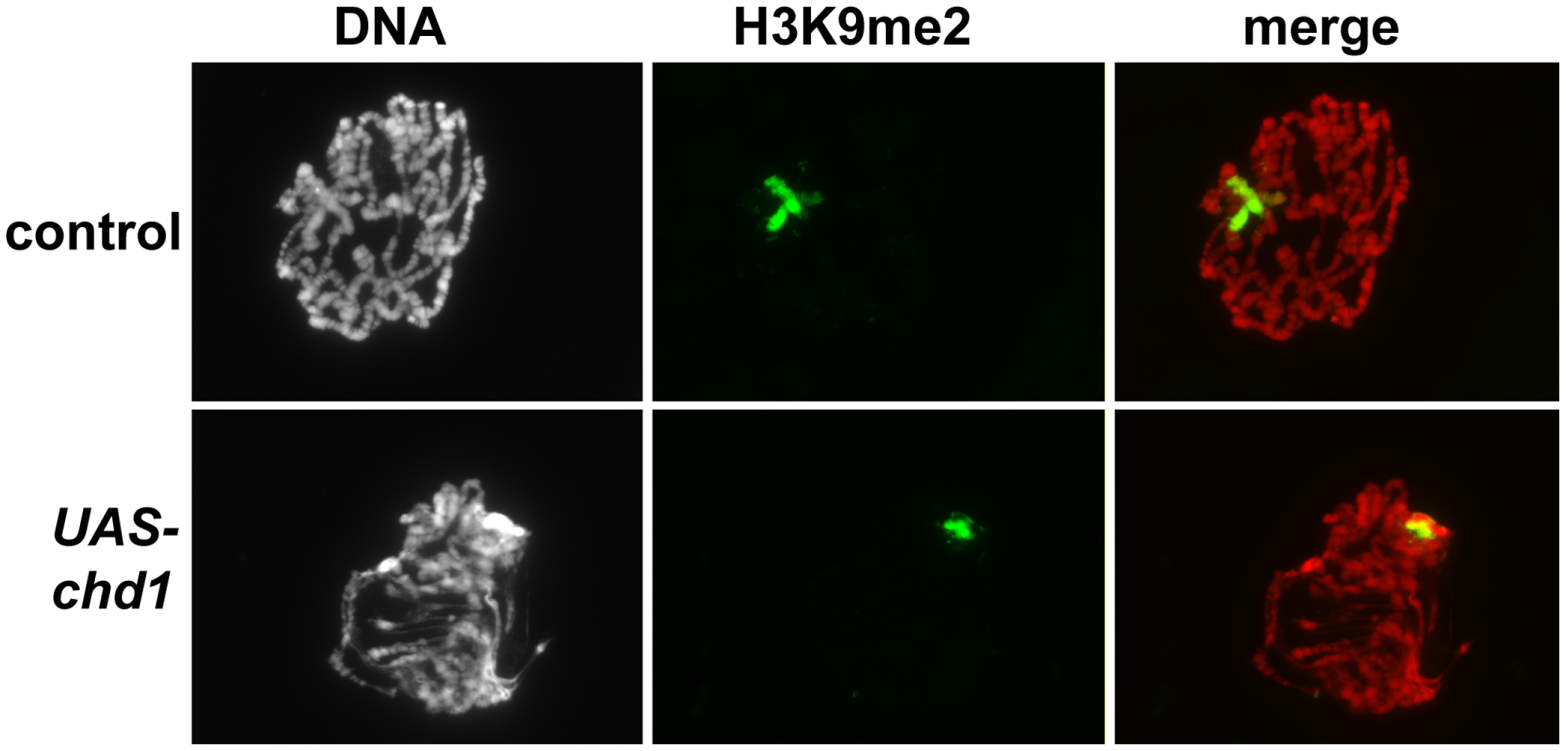
Over-expression of CHD1 results in a decrease in H3K9me2 levels. Polytene chromosomes derived from larvae over-expressing wild type *chd1* (*UAS-chd1*/*AB1-gal4*) show reduced H3K9me2 levels relative to controls (*UAS-lacZ/AB1-gal4*). Chromosomes were stained with DAPI (white in left panel, red in merge) and immunostained with anti-H3K9me2 (green) as described [40].

**Figure 7:**
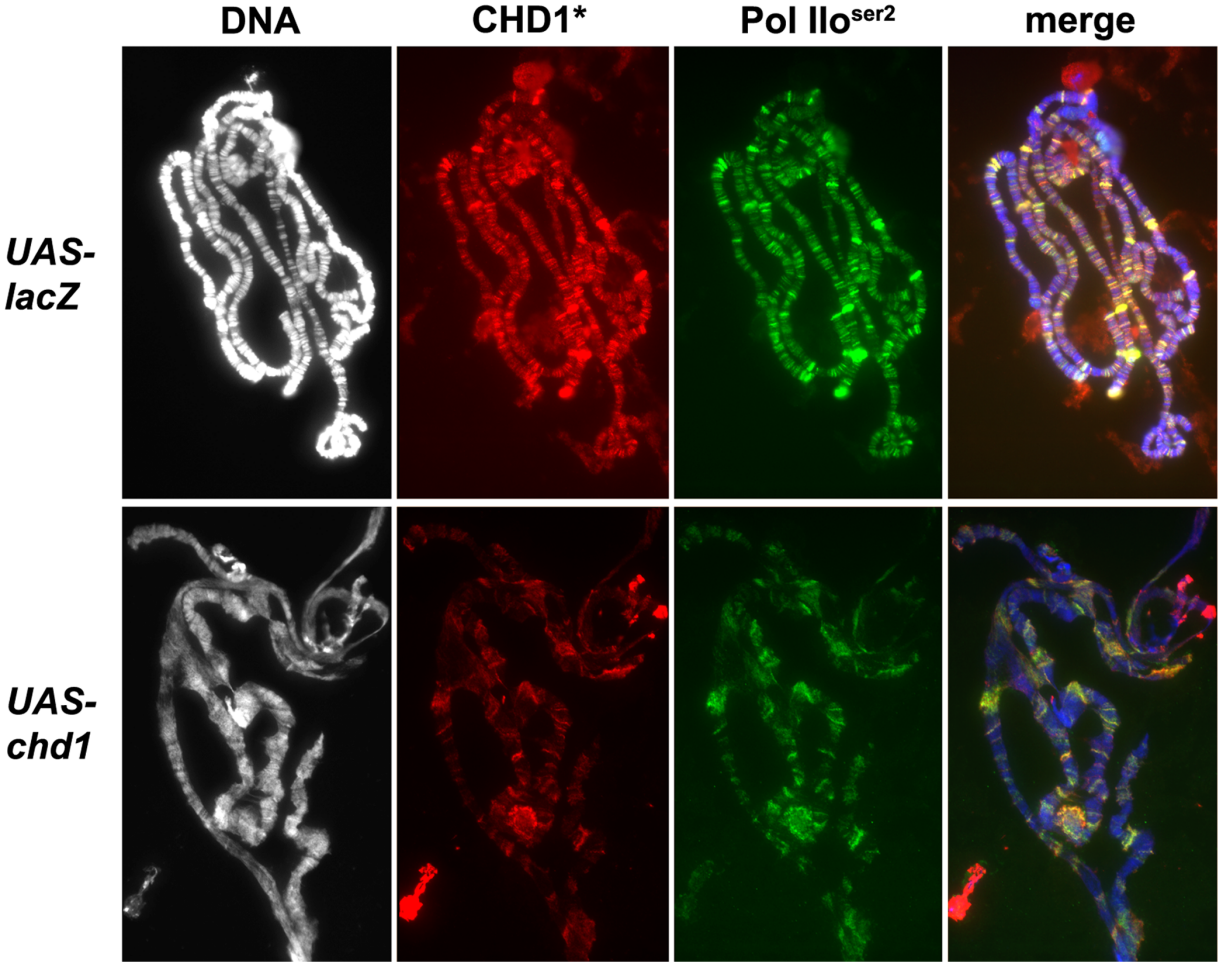
Over-expression of CHD1 does not affect elongating RNA Polymerase II. Polytene chromosomes derived from larvae over-expressing wild type CHD1 (*+*/*ey-gal4; UAS-chd1*/*+*) show similar levels of elongating RNA Pol II as compared to controls *(+/ey-gal4; UAS-lacZ/+*) relative to DAPI intensity despite significant defects in chromosome structure. Chromosomes are stained with DAPI (white in left panel, blue in merge) and immunostained with anti-Pol IIo^ser2^ (green) and anti-CHD1 (red) as described [7]. Asterisk indicates that exposure time for CHD1 was 56 msec for the control chromosomes and 7 msec for chromosomes over-expressing CHD1 in order to visualize co-localization of CHD1 with Pol II under both conditions.

To determine whether chromosomal defects resulting from over-expression of CHD1 are a consequence of a change in repressive chromatin, we examined the levels and distributions of HP1a on polytene chromosomes. Following over-expression of CHD1 we observed a range of HP1a patterns (Figure 5A). The bulk of the HP1a signal was dispersed into two to four locations, suggesting that the chromocenter was no longer intact. To confirm whether this phenotype is reproducible, the HP1a images were scored blind for the number of chromocenter-like puncta. We observed that indeed over-expression of CHD1 led to an increase of chromocenter-like puncta per single set of chromosomes (Figure 5B). While the levels of HP1a were variable, there was an overall reduction of the ratio of HP1a to DNA on chromosomes over-expressing CHD1 relative to controls (Figure 5C). Over-expression of the ATPase inactive form, CHD1^KR^, resulted in an intermediate phenotype, the HP1a signal was sometimes dispersed into more than one major site (Figures 5A and 5B), and the levels of HP1a were more moderately reduced (Figures 5A and 5C). To account for changes in overall protein levels due to compromised endoreplication, we calculated the ratio of HP1a to H3 on western blots loaded with an equal number of salivary glands. The ratio of total HP1a to total H3 was reduced 1.26-fold in glands over-expressing *chd1* relative to control glands, while over-expression of *chd1* resulted in a 5-fold decrease in the ratio of chromatin-bound HP1a to chromatin-bound histone H3 relative to controls (Figure 5D). Over-expression of *chd1^KR^* resulted in a 1.25-fold decrease in chromatin-bound HP1a relative to H3 (Figure 5D), suggesting that the ATPase domain of CHD1 plays an important role in counteracting repressive chromatin. Over-expression of *chd1* resulted in a reduction of H3K9me2 levels at the chromocenter and along the chromosomal arms (Figure 6). Although the structure of chromosomes derived from salivary glands over-expressing CHD1 can be severely disturbed, the H3K4me3 modification remained unchanged (Figure S7). Furthermore, RNA Pol IIo^ser2^ persisted, and the over-expressed CHD1 continued to co-localize with the elongating polymerase (Figure 7).

## Discussion

In this study we report that altering the levels of *Drosophila* CHD1 inversely affects HP1a and H3K9me2 levels and results in defects in the structure of polytene chromosomes. Heterochromatin is underrepresented in polytene chromosomes, however CHD1 is not found on mitotic chromosomes in flies [36], making polytene chromosomes an ideal setting in which to study the action of this conserved remodeler on higher order chromatin structure.

While most chromatin remodeling factors function in the context of large complexes, *Drosophila* CHD1 has not been found in a stable protein complex [37], and we observed that expression of an ATPase-inactive protein does not display phenotypes consistent with those expected from a dominant-negative allele. We note that over-expression of CHD1^KR^ often resulted in phenotypes that were intermediate between over-expression of wild type CHD1 and the LacZ negative control. If the mutant allele functions as a dominant-negative we would have expected phenotypes consistent with loss of function alleles–as is observed for other chromatin remodeling factors such as Brahma and ISWI [38]. If the CHD1^KR^ protein is simply inactive and inert, then we might have expected no phenotypes following over-expression. The intermediate phenotypes suggest that the CHD1^KR^ protein may retain some function. Thus, we propose that the CHD1 remodeler maintains chromosome structure in the absence of stoichiometric, stable protein partners, and that the ATPase domain may be dispensable for some functions.

Loss of the CHD1 chromatin remodeler resulted in an increase in the levels of HP1a and H3K9me2 on polytene chromosomes with no global change in the euchromatic mark H3K4me3. Surprisingly, the increase in heterochromatic elements correlates with polytene chromosomes that often appear hypocondensed or even puffy (see for example, Figure S1A). Analysis of DAPI levels suggests that DNA replication is not impaired in salivary glands lacking CHD1, suggesting that a change in the quantity of DNA is not responsible for the observed defects. Chd1 antagonizes heterochromatic elements in embryonic stem cells in mice [25]; our findings indicate that this function of CHD1 extends to other organisms as well as to differentiated cells. It is not known whether the changes in HP1a and H3K9me2 are a cause or an effect of changes in polytene structure, or if they are a completely distinct consequence of loss of *chd1*.

It is currently unclear how CHD1 alters levels of HP1 and H3K9me2, particularly as CHD1 is not found in high levels at telomeres or the heterochromatic chromocenter, but instead is enriched in DAPI interbands and transcriptionally active genes [8], [20]. It is possible that CHD1 is functioning indirectly, for example by repressing expression of genes that are responsible for heterochromatin. In support of this model, we observe a modest increase in cellular HP1a by western blot of salivary glands (relative to H3) following loss of *chd1*. However, a recent microarray analysis of gene expression in flies revealed that *Su(var)205* (which encodes HP1a) and *Su(var)3-9* (which encodes a H3K9 methyltransferase) were not among the 602 genes that were up-regulated or the 421 genes that were down-regulated in *chd1* mutant larvae [39]. Rather than genes involved in chromatin structure, Sebald et al. observed an enrichment of genes important for a broad range of responses to a variety of different types of stresses [39]. As a result of these observations, we favor a model in which CHD1 directly alters chromatin structure.

Two other chromatin remodeling factors also affect polytene chromosome structure when mis-regulated: ISWI [40]–[42] and dMi-2 [10]. Due to antagonism of ISWI function by H4K16 acetylation on the dosage-compensated male X chromosome, *ISWI* mutant males display a striking puffy X phenotype [43], a phenotype we have not observed in *chd1* mutant larvae or in larvae over-expressing *chd1*. Over-expression of a dominant-negative allele of *ISWI* (an ATPase mutant) leads to more globally disrupted polytene chromosomes [40], [42]. Loss of ISWI function results in a loss of linker histone H1 on polytene chromosomes [40], [42], and it is possible that CHD1 is similarly altering H1 localization. Over-expression of *dMi-2* results in polytene chromosomes that lack a precise banding pattern when fixed, are larger than control chromosomes when viewed live, and display a reduction in the stability of cohesin binding [10]. Given the localization of both CHD1 and cohesin to active genes [8], [10], it will be important to determine whether the CHD1 affects the binding or stability of this intriguing protein.

CHD1 is a major player in nucleosome dynamics. Unlike most chromatin remodeling factors, CHD1 possesses the ability to function with a histone chaperone to facilitate nucleosome assembly *in vitro* [37]. *In vivo,* CHD1 is important for the replication-independent assembly of H3.3-containing nucleosomes on paternal chromosomes following fertilization in *Drosophila* [28], and is required for the deposition and/or stability of H3.3 in salivary gland nuclei [29]. As the H3.3 histone variant is deficient in H3K9me2 [33], a reduction in H3.3 deposition in *chd1* mutants could lead to an increase in this heterochromatin mark. A role for CHD1 in nucleosome dynamics is not limited to flies. In both *S. pombe* and *S. cerevisiae,* Chd1 proteins are critical for nucleosome positioning and occupancy over gene bodies [24], [44], [45]. In *S. cerevisiae*, Chd1 promotes nucleosome turnover at the 5′ ends of genes and nucleosome stability at 3′ ends of active genes [29], [46]. Mammalian cells have two proteins similar to *Drosophila* CHD1 (called CHD1 and CHD2); CHD2 deposits H3.3 in the promoters of MyoD target genes in myoblasts prior to their expression [47]. Our study raises the question as to whether the action of CHD1 in regards to nucleosome positioning or composition across genes might have consequences for the maintenance of correct levels of HP1a and H3K9me2 and global chromosome structure.

## Methods

### *Drosophila* Stocks and Crosses

Flies were raised on standard cornmeal-molasses-yeast-agar medium containing Tegosept and propionic acid at 25°C unless otherwise indicated. The Vienna *Drosophila* RNAi Center *RNAi-chd1* flies (*VDRC26277* and *VDRC103640*) and *RNAi-spt5* (*VDRC19793*) (http://stockcenter.vdrc.at/) were crossed to GAL4 drivers, and larvae expressing hpRNA were raised at 29°C. Homozygous *chd1^4^* and *chd1^5^* null mutant larvae [15] were raised at 18°C. Control flies *w; P[w^+mC^, UAS-lacZ.Exel]2* and *w^1118^; P[UAS-EGFP]34/TM3, Sb^1^* as well as the GAL4 drivers y^1^ w*; P[Act5C-GAL4]17b15O1/TM6B, Tb^1^ (referred to as *Actin-GAL4*) and y^1^ w*; P[w^+mW.hs^ = GawB]AB1 (referred to as *AB1-GAL4*) were obtained from Bloomington *Drosophila* Stock Center (http://flystocks.bio.indiana.edu/). The *eyeless-GAL4* driver was a gift from John Tamkun (University of California, Santa Cruz) [48]. Lines over-expressing *chd1* and *chd1^KR^*, *w; P[w^+^, UAS-chd1^+^]126* and *w; P[w^+^, UAS-chd1^KR^]88*, were constructed from the *chd1* cDNA and were a generous gift from Helen McNeill (Samuel Lunenfeld Research Institute, Mount Sinai Hospital).

### Analysis of Polytene Chromosomes

Immunostaining of polytene chromosomes from third instar larvae were performed by several different formaldehyde fixation methods [7], [49]–[51] or by citric acid fixation [40]. The use of several fixation protocols for each antibody ensured that our results were not an artifact from one particular method, a well-documented concern [52]. For each protocol, slides with squashed chromosomes were blocked and incubated with primary antibodies overnight at 4°C. Mouse IgG anti-HP1a antibody (C1A9 Developmental Studies Hybridoma Bank, University of Iowa [53]) was used at 1∶50, rabbit anti-CHD1 [15] was used at 1∶250, mouse IgM anti-Pol IIo^ser2^ (H5) (Covance) was used at 1∶50. Rabbit antibodies directed against H3K9me2 and H3K4me3 (Millipore #07-212and #05-745R respectively) were used at 1∶100. Slides were washed and incubated in the appropriate secondary antibodies diluted at 1∶200 (Jackson ImmunoResearch) for 1 h at room temperature, then washed and mounted in Vectashield containing DAPI (Vector Laboratories). A minimum of three slides of each control and experimental genotypes were prepared at the same time and photographed using identical exposure times using an Olympus IX81 microscope with a CoolSNAP HQ^2^ camera (Photometrics) and ImagePro6.6 image software. Fig. 7 was photographed on a Zeiss Axioskop2 plus microscope with an Axioplan HRm camera and Axiovision 4 imaging software (Carl Zeiss, Germany). Five to eight chromosomal spreads were chosen from each slide for imaging, and images were processed identically using Adobe Photoshop CS3. To quantify immunofluorescence signals on polytene chromosomes relative to DAPI intensity, we developed a program in Matlab 7.4.0. The code inputs batches of images, each with up to three fluorescent channels, and allows the user to effectively remove non-chromosomal antibody staining by applying a mask that only exposes the polytene chromosomes. To facilitate use, the program has a Java-based graphical user interface. The program can be downloaded from http://faculty.jsd.claremont.edu/jarmstrong/fquant/index.html.

### Western Blot Analysis

Salivary gland chromatin extracts and total protein extracts were prepared from isolated salivary glands of third instar larvae as described [40]. The equivalent of five salivary glands was loaded into each lane, and proteins were resolved by 15% SDS-PAGE and transferred onto nitrocellulose membrane. Membranes were hybridized with primary antibodies anti-HP1a at 1∶1000 (C1A9 Developmental Studies Hybridoma Bank, University of Iowa) and anti-H3 (Abcam, 1∶30,000) and secondary antibodies goat anti-mouse-HRP (1∶5000, BioRad) and goat anti-rabbit-HRP (1∶20,000, BioRad), and were detected with Supersignal West Dura (Pierce). Multiple exposures of film were scanned and quantified using ImageJ.

## Supporting Information

Figure S1 **Loss of CHD1 by hpRNA results in polytene chromosomes with a disrupted structure.** (A) Oregon R, a wild type strain (control) and *w; VDRC26277/+; Act5c-GAL4/+,* (*RNAi-*chd1) DNA is stained with DAPI. (B) Location of *chd1* hpRNA sequences *VDRC26277* (red) and *VDRC103640* (green) [30]. Image created in GenePalette [54].
(TIF)

Figure S2 **Expression of hpRNA directed against** ***spt5*** **does not affect chromosome structure.** *lacZ* and *RNAi-spt5* (VDRC19793) were expressed in salivary glands using the *AB1-gal4* driver. DNA is stained with DAPI.
(TIF)

Figure S3 **Loss of CHD1 does not alter DNA content, while over-expression of CHD1 results in a decrease in DNA levels.** (A) DNA levels of intact polytene squashes (containing a complete complement of chromosomes) derived from *UAS-lacZ/AB1-gal4* (N = 19), *VDRC26277/+; AB1-gal4/+* (N = 8) and *VDRC103640/+; AB1-gal4/+* (control, N = 13). (B) DNA levels of intact polytene squashes (containing a complete complement of chromosomes) derived from *UAS-lacZ/AB1-gal4* (control, N = 17), *UAS-chd1/AB1-gal4* (N = 6), and *UAS-chd1^KR^*/*AB1-gal4* (N = 6). Experiments shown in (A) and (B) are each representative of several independent experiments. (C) Polytenes prepared from *UAS-chd1/AB1-gal4* larvae (*UAS-chd1*) are malformed; *UAS-lacZ/AB1-gal4* (control) and the majority of *UAS-chd1^KR^/AB1-gal4* larvae show normal morphology. DNA is stained with DAPI, chromosomes prepared as described [49].
(TIF)

Figure S4 **Loss of CHD1 results in an increase in HP1a on polytene chromosomes.** Chromosomes derived from *UAS-lacZ/AB1-gal4* and *VDRC103640/+; AB1-gal4/+* (*RNAi-chd1*) larvae were immunostained with anti-HP1a (green) as described [51], DNA is stained with DAPI (white in left panel, red in merge).
(TIF)

Figure S5 **The ATPase domain of CHD1 is important for its action on chromosome structure.** Chromosomes derived from salivary glands over-expressing an ATPase inactive form of CHD1 (*UAS-chd1^KR^/AB1-gal4)* appear normal in structure, similar to control chromosomes derived from *UAS-gfp/AB1-gal4* larvae and unlike chromosomes derived from *UAS-chd1/AB1-gal4* larvae. To visualize the banding patterns of CHD1 on chromosomes over-expressing the protein and to compare relative expression levels, the exposure time for the CHD1 antibody is lower than what is normally used. Using this short exposure time, CHD1 is not visible on control chromosomes. Control chromosomes were therefore processed independently in Photoshop in order to visualize CHD1 (inset image).
(TIF)

Figure S6 **Quantification of CHD1 bound to chromosomes.** Polytene chromosomes from *UAS-chd1*/*AB1-gal4* glands show 4.7 fold more CHD1 bound to chromosomes as compared to control glands expressing GFP. Over-expression of the ATPase mutant form of *chd1* results in a 4.0 fold increase in CHD1 levels. Note that the antibody used cannot distinguish between endogenous CHD1 and over-expressed CHD1 or CHD1^KR^.
(TIF)

Figure S7 **Levels of H3K4me3 are unaffected by over-expression of CHD1.** Polytenes derived from *UAS-gfp/AB1-gal4* (control) larvae and *UAS-chd1/AB1-gal4* larvae show similar levels of the transcriptionally active mark H3K4me3. Chromosomes were stained with DAPI (white in left panel, red in merge) and immunostained with anti-H3K4me3 (green) as described [40].
(TIF)
