# Supplementary figures and images for: The Drosophila melanogaster CHD1 Chromatin Remodeling Factor Modulates Global Chromosome Structure and Counteracts HP1a and H3K9me2
Source: PLoS One. 2013 Mar 22;8(3):e59496. doi: 10.1371/journal.pone.0059496 (PMC3606111; doi:10.1371/journal.pone.0059496)

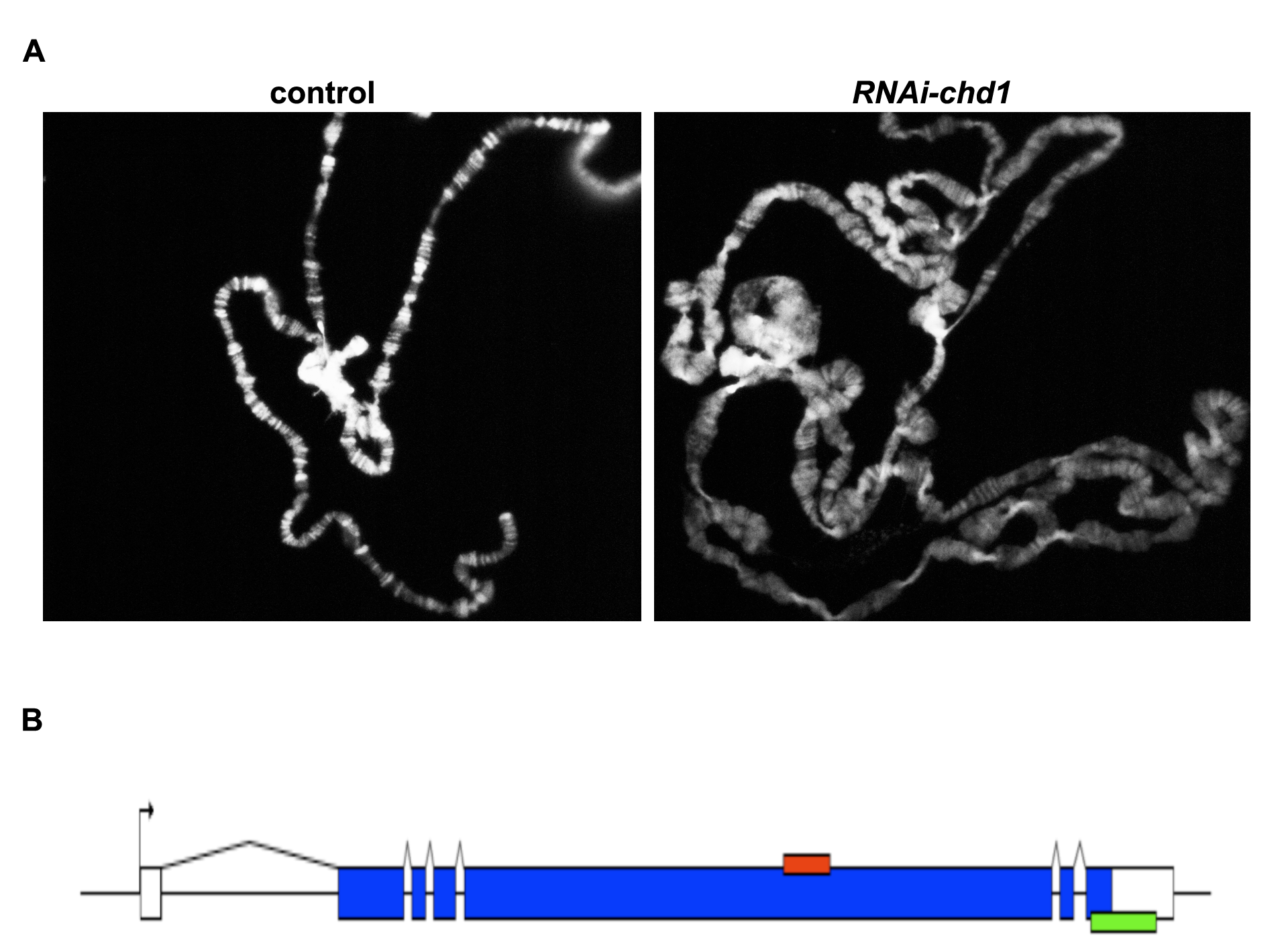

Supplement: Figure S1 — Loss of CHD1 by hpRNA results in polytene chromosomes with a disrupted structure. (A) Oregon R, a wild type strain (control) and w; VDRC26277/+; Act5c-GAL4/+, (RNAi-chd1) DNA is stained with DAPI. (B) Location of chd1 hpRNA sequences VDRC26277 (red) and VDRC103640 (green) [30]. Image created in GenePalette [54]. (TIF) [file pone.0059496.s001.tif]

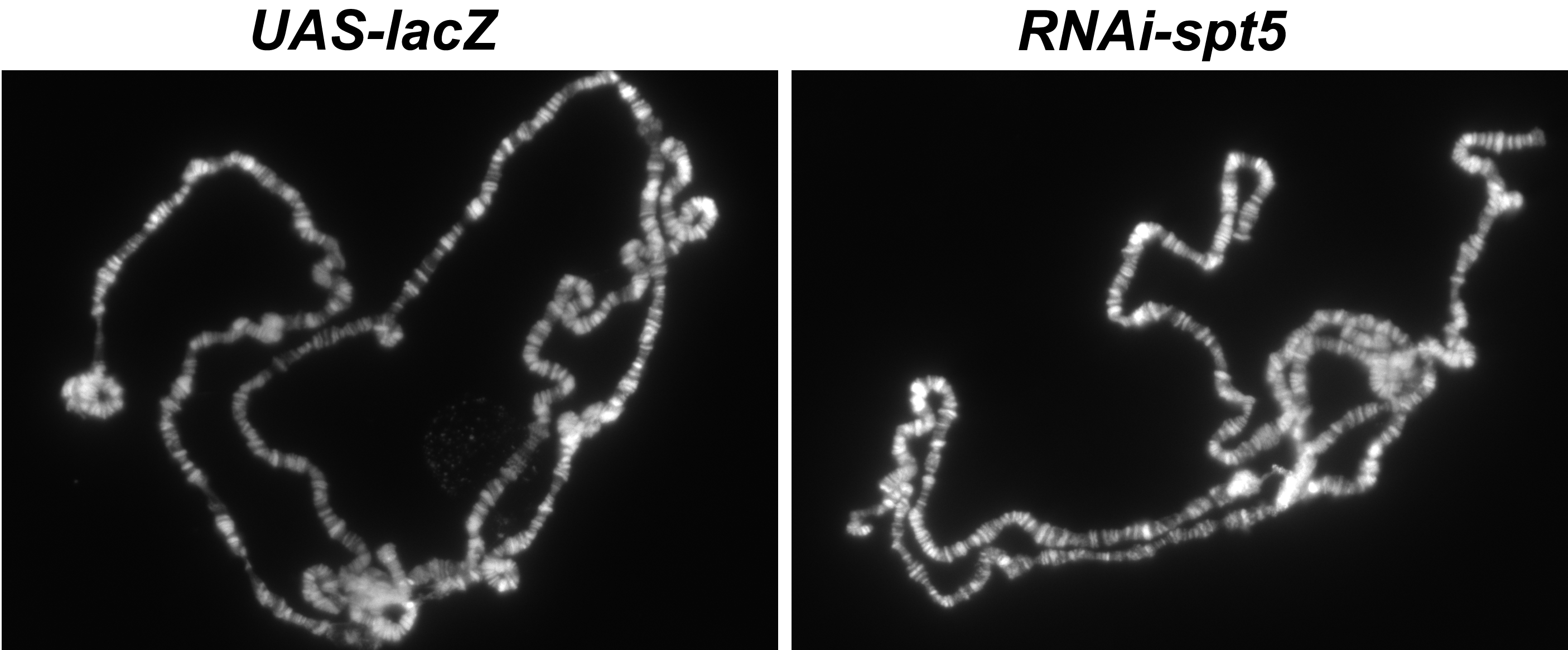

Supplement: Figure S2 — Expression of hpRNA directed against spt5 does not affect chromosome structure. lacZ and RNAi-spt5 (VDRC19793) were expressed in salivary glands using the AB1-gal4 driver. DNA is stained with DAPI. (TIF) [file pone.0059496.s002.tif]

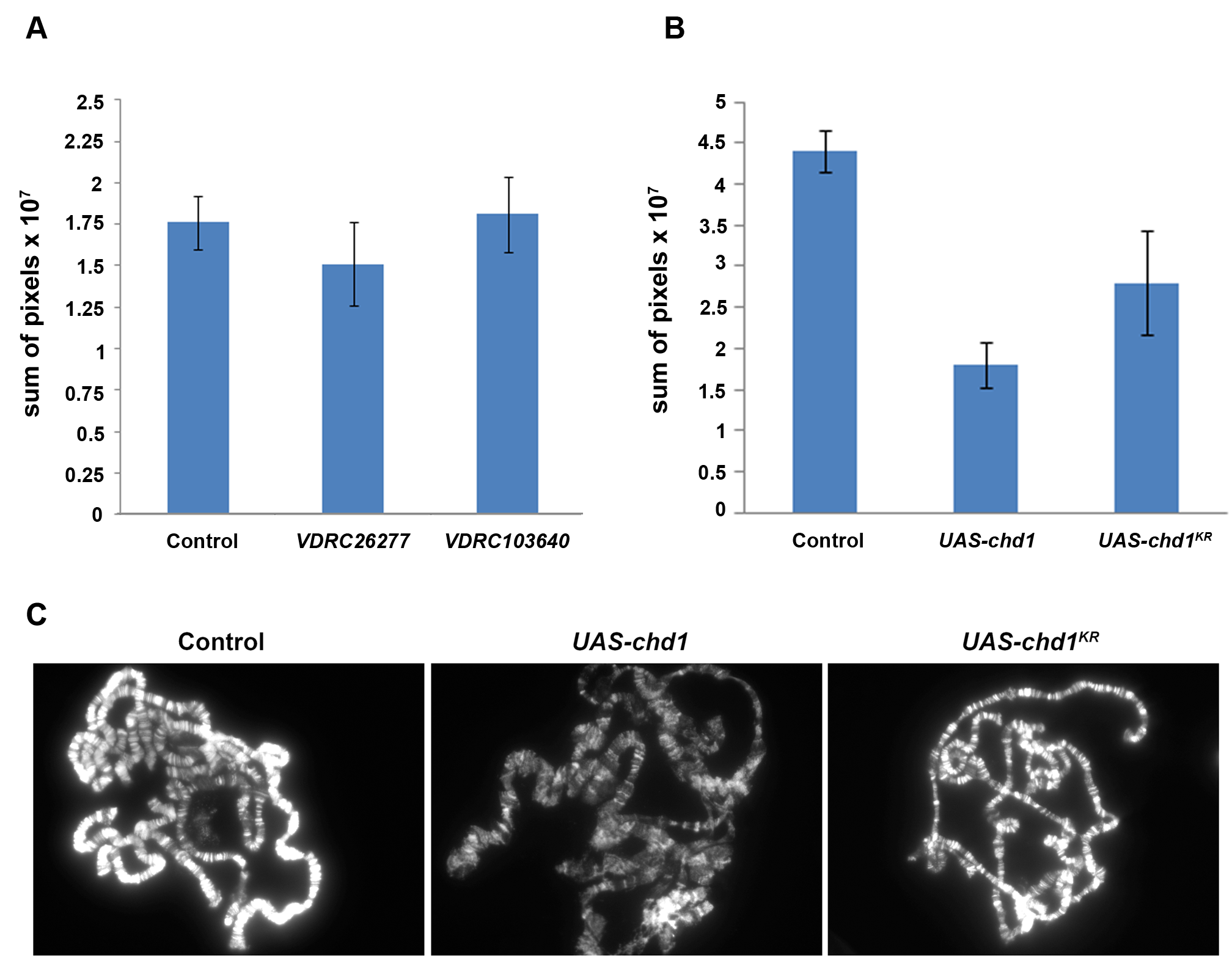

Supplement: Figure S3 — Loss of CHD1 does not alter DNA content, while over-expression of CHD1 results in a decrease in DNA levels. (A) DNA levels of intact polytene squashes (containing a complete complement of chromosomes) derived from UAS-lacZ/AB1-gal4 (N = 19), VDRC26277/+; AB1-gal4/+ (N = 8) and VDRC103640/+; AB1-gal4/+ (control, N = 13). (B) DNA levels of intact polytene squashes (containing a complete complement of chromosomes) derived from UAS-lacZ/AB1-gal4 (control, N = 17), UAS-chd1/AB1-gal4 (N = 6), and UAS-chd1KR/AB1-gal4 (N = 6). Experiments shown in (A) and (B) are each representative of several independent experiments. (C) Polytenes prepared from UAS-chd1/AB1-gal4 larvae (UAS-chd1) are malformed; UAS-lacZ/AB1-gal4 (control) and the majority of UAS-chd1KR/AB1-gal4 larvae show normal morphology. DNA is stained with DAPI, chromosomes prepared as described [49]. (TIF) [file pone.0059496.s003.tif]

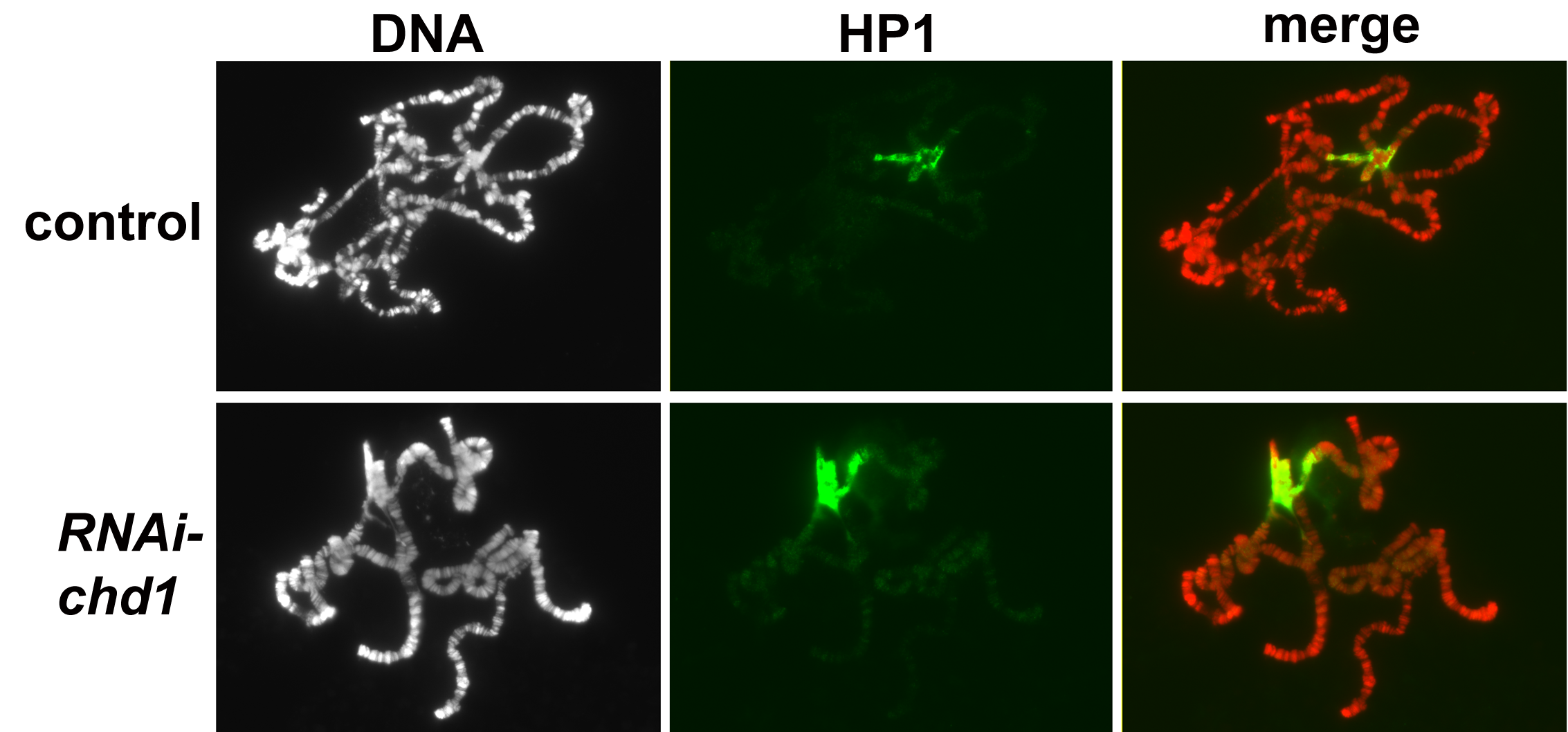

Supplement: Figure S4 — Loss of CHD1 results in an increase in HP1a on polytene chromosomes. Chromosomes derived from UAS-lacZ/AB1-gal4 and VDRC103640/+; AB1-gal4/+ (RNAi-chd1) larvae were immunostained with anti-HP1a (green) as described [51], DNA is stained with DAPI (white in left panel, red in merge). (TIF) [file pone.0059496.s004.tif]

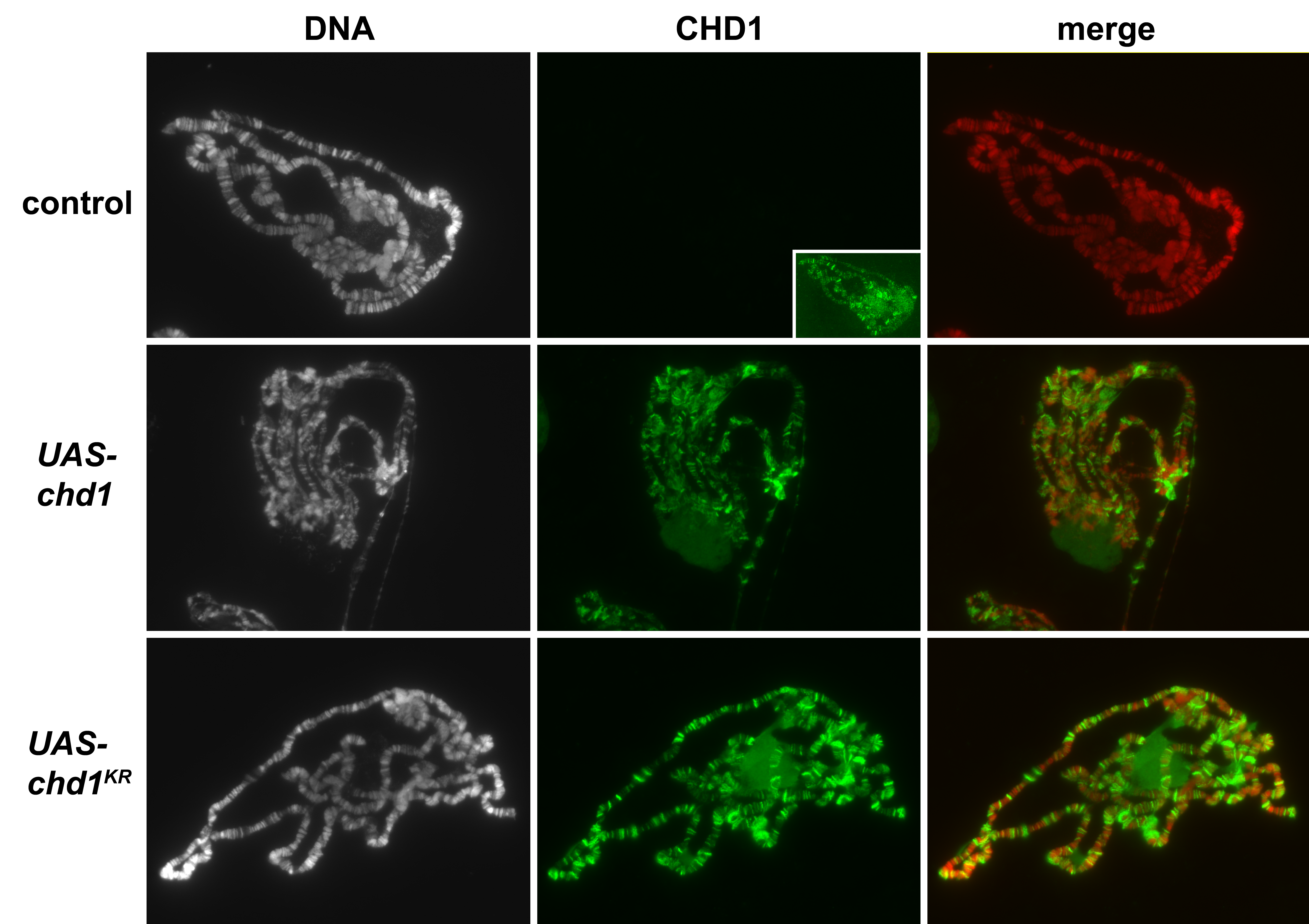

Supplement: Figure S5 — The ATPase domain of CHD1 is important for its action on chromosome structure. Chromosomes derived from salivary glands over-expressing an ATPase inactive form of CHD1 (UAS-chd1KR/AB1-gal4) appear normal in structure, similar to control chromosomes derived from UAS-gfp/AB1-gal4 larvae and unlike chromosomes derived from UAS-chd1/AB1-gal4 larvae. To visualize the banding patterns of CHD1 on chromosomes over-expressing the protein and to compare relative expression levels, the exposure time for the CHD1 antibody is lower than what is normally used. Using this short exposure time, CHD1 is not visible on control chromosomes. Control chromosomes were therefore processed independently in Photoshop in order to visualize CHD1 (inset image). (TIF) [file pone.0059496.s005.tif]

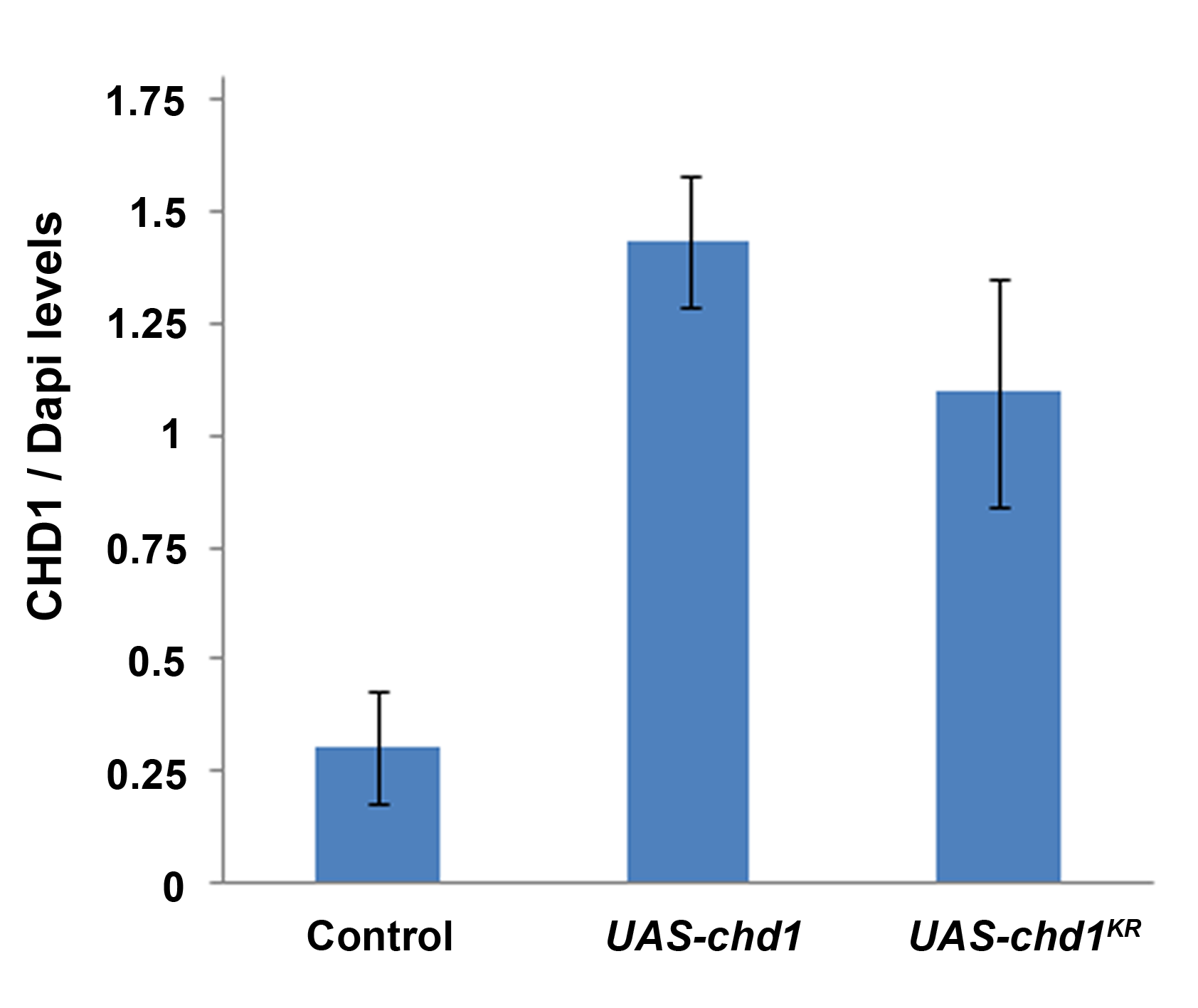

Supplement: Figure S6 — Quantification of CHD1 bound to chromosomes. Polytene chromosomes from UAS-chd1/AB1-gal4 glands show 4.7 fold more CHD1 bound to chromosomes as compared to control glands expressing GFP. Over-expression of the ATPase mutant form of chd1 results in a 4.0 fold increase in CHD1 levels. Note that the antibody used cannot distinguish between endogenous CHD1 and over-expressed CHD1 or CHD1KR. (TIF) [file pone.0059496.s006.tif]

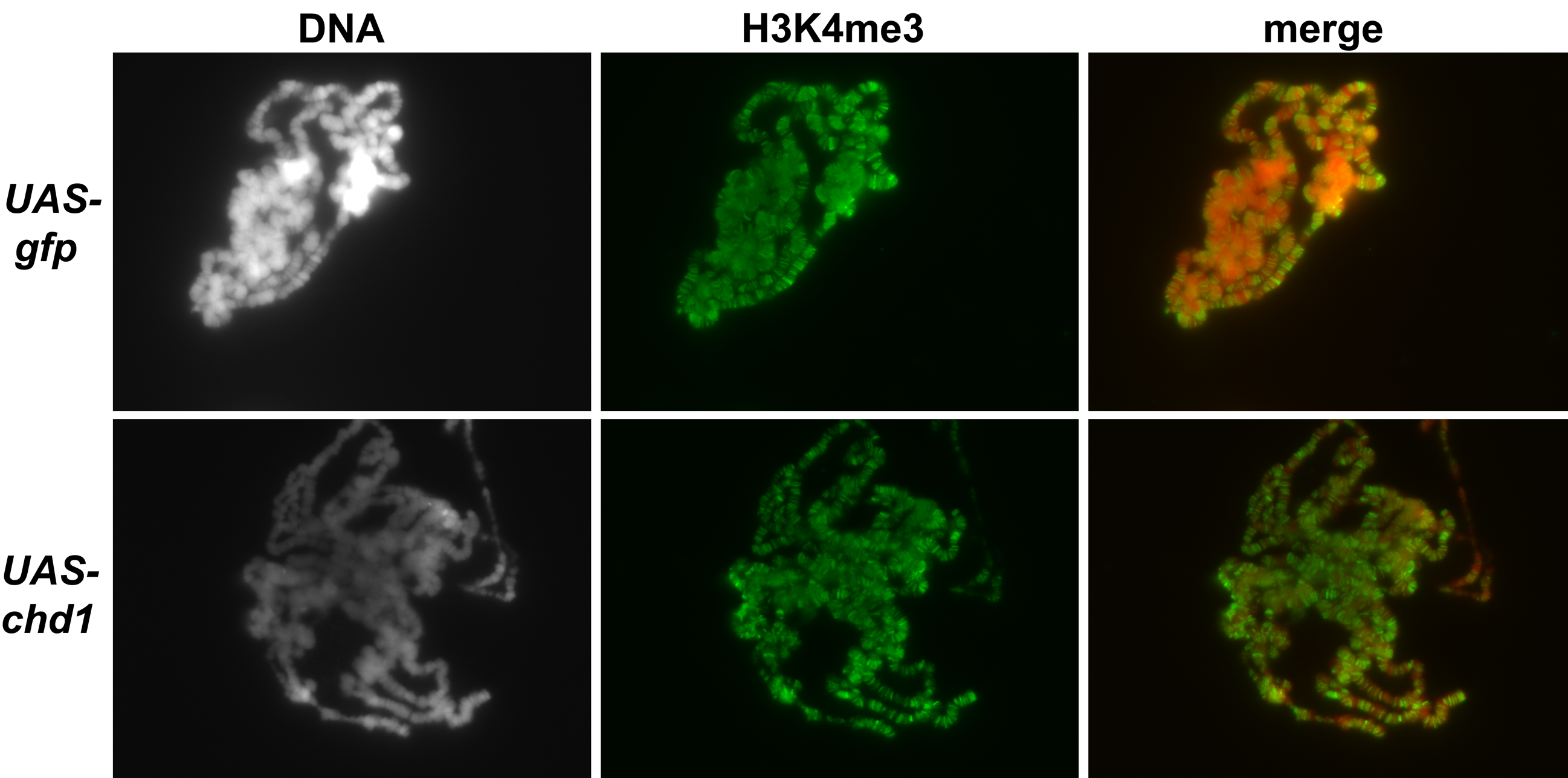

Supplement: Figure S7 — Levels of H3K4me3 are unaffected by over-expression of CHD1. Polytenes derived from UAS-gfp/AB1-gal4 (control) larvae and UAS-chd1/AB1-gal4 larvae show similar levels of the transcriptionally active mark H3K4me3. Chromosomes were stained with DAPI (white in left panel, red in merge) and immunostained with anti-H3K4me3 (green) as described [40]. (TIF) [file pone.0059496.s007.tif]
